# Understanding Parents' Experiences of Using a Portion Guide for Young Children: A Qualitative Study

**DOI:** 10.1111/mcn.70151

**Published:** 2025-12-13

**Authors:** Mira Malmberg, Rebecca Lang, Rana Conway

**Affiliations:** ^1^ Research, Department of Behavioural Science and Health University College London London UK; ^2^ HENRY, Elm Place Eynsham Oxfordshire UK

**Keywords:** early years, guidance, portion size, preschool children, qualitative, toddler

## Abstract

Serving children larger portion sizes is associated with higher energy intake and can contribute to childhood obesity. Parents of young children report being open to receiving portion guidance. However, the perspective of parents who have received a portion guide is not well understood. The current study aimed to (i) understand how parents provided with an age‐appropriate portion guide use it to guide feeding behaviour and (ii) assess the value of making age‐appropriate portion guidance more widely available. In‐depth, semi‐structured interviews were conducted with 15 parents of young children (1–4 years) who had received an age‐appropriate portion guide. Interviews were analysed using reflexive thematic analysis and four themes were developed: (i) guidance is appreciated but has a limited impact on portion sizes, (ii) portions are usually determined by other factors, (iii) the guide is still useful, just not as intended and (iv) when and how guides are delivered influences acceptability. Parents were receptive to feeding guidance from trusted sources. The portion guide was seen as a useful resource for maintaining balance in children's diets along with limiting less healthy foods, but was not used directly to guide the portions parents served. Several aspects of the guide were seen as impractical and unrealistic, and portions served were mainly determined using parent‐ and child‐led strategies. Results suggest that portion guides might be less useful for parents' portioning practices than previously assumed. However, portion guides are still appreciated by parents and positively influence other aspects of feeding behaviour.

## Summary

- Parents appreciated being provided with an age‐appropriate portion guide for young children, but found it impractical to use at busy mealtimes.
- Instead, parents relied on a range of parent‐ and child‐driven strategies to determine portion sizes.
- Parents believed the food groups and example foods shown on the portion guide helped them to balance their children's diets and provided ideas for meals.
- Age‐appropriate portion guides may be less useful for parents than assumed and example foods shown on feeding guides need to be carefully selected.

## 1 Introduction

In the 21st century, childhood obesity has emerged as a significant public health challenge (Nisar 2018; Sahoo et al. 2015). More than one in five children in England are living with overweight or obesity when they start primary school (aged 4–5 years), with the prevalence of obesity being more than twice as high among children in the most deprived areas compared to the least deprived regions (NHS 2025). Research suggests early weight development is highly predictive of overweight or obesity in later childhood and adulthood. For example, analysis of data from more than 50,000 participants indicates that 90% of children experiencing obesity at age 3 years continue to experience overweight or obesity in adolescence (Geserick et al. 2018). As the adverse physical and psychological consequences of childhood obesity persist into adulthood, it is essential to promote healthy weight development in the early years (Rankin et al. 2016; Reilly and Kelly 2011).

Many food preferences and eating habits observed in adulthood develop during early childhood and associations have been shown between parental feeding behaviours and children's eating behaviours (Murray 2017; Nicklaus 2017; Birch and Doub 2014; Ventura and Worobey 2013). Infants are generally considered good at self‐regulating energy intake in the first year of life, with this ability diminishing in early childhood (Brugaillères et al. 2019; Fox et al. 2006). Although some children are genetically inclined to experience poorer satiety responses, which puts them at increased risk of consuming larger portions when offered them (Syrad et al. 2016). Parental pressure to eat can potentially override a child's internal satiety cues and increase the risk of overeating (Hetherington and Blundell‐Birtill 2018), while responsiveness to a child's hunger and satiety cues can enhance children's self‐regulation (DiSantis et al. 2011).

Parents are often more concerned about young children eating too little than too much (Daniels et al. 2012), but the Scientific Advisory Committee on Nutrition [SACN] (2023) points to larger portions potentially contributing to high energy intakes. Healthcare providers report parents overestimating appropriate portion sizes, particularly for high‐sugar foods (Heller et al. 2021). This is problematic as children have been found to consume more energy when they are served larger portions, which is known as the ‘portion size effect’ (Birch et al. 2015; Small et al. 2013, Zlatevska et al. 2014), and evidence suggests this is an important contributor to childhood adiposity (Dobrescu et al. 2025; Johnson et al. 2014; McConahy et al. 2004; Syrad et al. 2016). This effect is amplified when meals include energy‐dense foods, creating a phenomenon known as ‘double trouble’ (Kling et al. 2016). Studies with children aged 3–5 years indicate that when they are served larger portions, they do not adequately adjust later energy intake (Fisher et al. 2007; Smethers et al. 2019). Therefore, serving children age‐appropriate portions is critical for the prevention of childhood overweight and obesity.

Parents report using a variety of strategies when deciding how much to feed their children, often relying on instincts and previous experiences (Johnson et al. 2015; Philippe et al. 2021), knowledge (Carnell et al. 2011; Jacquier et al. 2016), and visual aids, such as bowls or packaging sizes (Kairey et al. 2018; Porter et al. 2023). Many parents report being unsure of age‐appropriate portion sizes for young children (Curtis et al. 2017; Tang et al. 2020; Reale et al. 2019) and SACN highlight the need to develop and communicate age‐appropriate portion guidance (Scientific Advisory Committee on Nutrition [SACN] 2023). Despite many portion guidance tools being available in the United Kingdom (Porter et al. 2020), none are routinely provided to parents, and parents may be unaware of them (Porter et al. 2023). However, parents of young children in several studies have indicated they would like, and would use, an age‐appropriate portion guide (Martin‐Biggers et al. 2015; Reale et al. 2019; Philippe et al. 2021).

**Figure 1:**
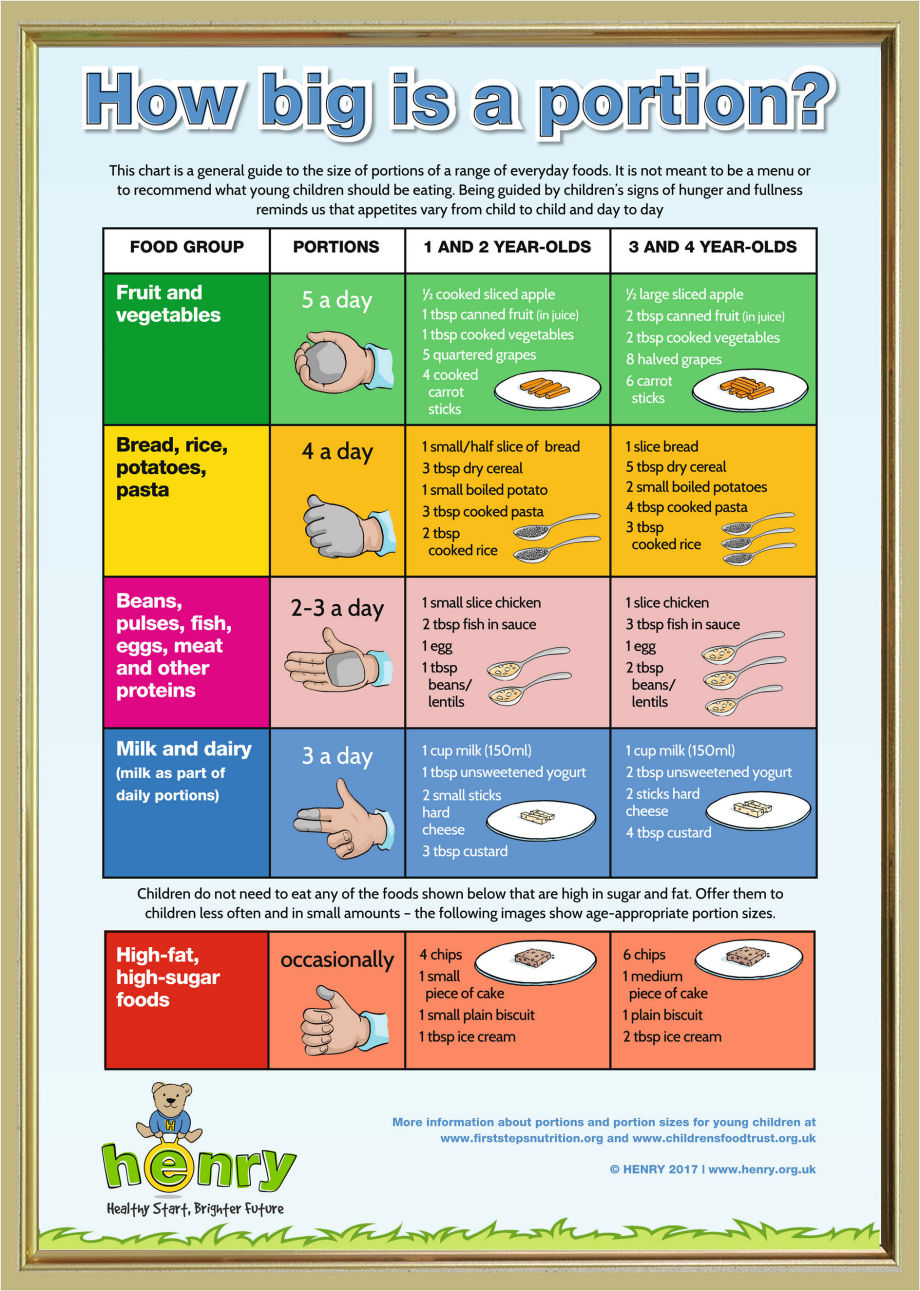
HENRY portion guide: How big is a portion?

The UK charity HENRY (Health, Exercise, Nutrition for the Really Young) provides an age‐appropriate portion guide (Figure 1), alongside other resources, to parents attending their Healthy Families Right from the Start (HFRFTS) programme. HFRFTS is a widely commissioned programme, particularly in areas of the United Kingdom experiencing higher levels of deprivation, which supports responsive feeding and healthy eating in infants and preschool children (Willis et al. 2016, Department of Health and Social Care 2017). Analysis of survey data collected before and after attending the programme indicates improvements in parental feeding practices (Willis et al. 2014, 2016) and associated reductions in early childhood overweight and obesity rates (Rudolf et al. 2019), although the role of the portion guide per se in the success of the programme has not been assessed.

While the literature suggests that parents of young children would use a portion guide if it were provided, the perspective and experiences of parents who have received such a guide are not well understood. This study aimed to (i) understand how parents and main caregivers of young children provided with the HENRY portion guide use it to guide feeding behaviour and (ii) assess the value of making age‐appropriate portion guides more widely available to families.

## 2 Methods

### 2.1 Participants

Eligibility criteria included: > 18 years old, living in England, parent or main carer (hereafter referred to as ‘parents’ or ‘participants’ for brevity) of a child aged 1–4 years who had received the ‘How big is a portion?’ resource (Figure 1). HENRY programme coordinators from eight regions in England disseminated study information to parents in workshops and via social media and newsletters. Interested parents completed a screening survey to assess eligibility and suitable participants were contacted via email and provided with an information sheet and consent form.

The study aimed to recruit 15–20 participants, which was considered appropriate, based on the principles of information power, as the data were focused and participants had rich experiences relevant to the research question (Malterud et al. 2016). Purposive sampling was used to select participants from underrepresented ethnic and socioeconomic groups. The Index of Multiple Deprivation (IMD; Ministry of Housing, Communities and Local Government 2019) was used to assess level of deprivation of participants' residential areas, while socioeconomic position was self‐reported using the Social Grade classification (Office for National Statistics 2021). Participants received a £25 shopping voucher.

### 2.2 Qualitative Interviews

A semi‐structured interview schedule (Supporting Information S1: Appendix A) was developed in collaboration with HENRY and informed by previous literature. Interviews were conducted online by M.M., a female researcher who does not have children and was previously unknown to participants. It was communicated to parents that M.M. was not a part of HENRY, and they were encouraged to answer questions honestly even if they were critical of HENRY. Interview questions focused on four topics: (i) general decisions about feeding, (ii) perceptions of using the portion guide, (iii) feelings about specific aspects of the guide and (iv) preferred modality of portion guidance. Interviews were video recorded and transcribed verbatim using Microsoft Teams. M.M. wrote field notes after each interview to record initial ideas. Transcripts were reviewed and edited for accuracy and to remove identifying details.

### 2.3 Analysis

**Figure 2:**
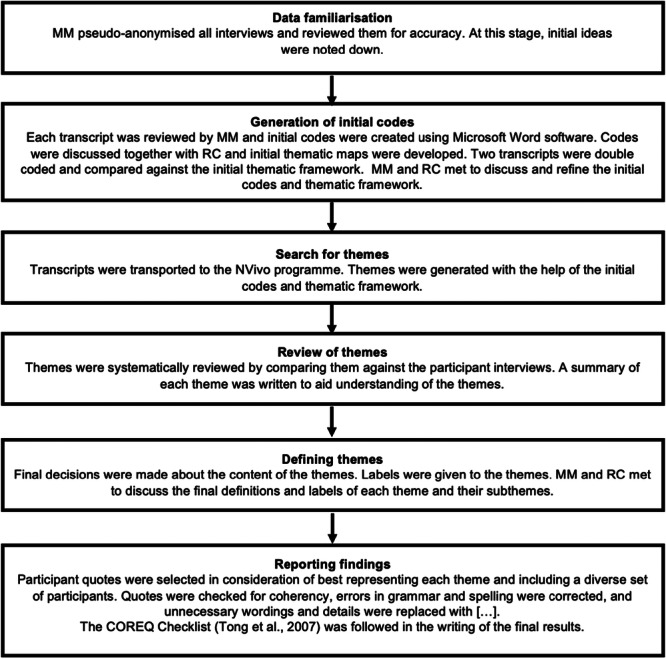
Overview of qualitative analysis process.

Reflexive thematic analysis (TA) was used by M.M. as the methodology is suitable for investigating the shared experience of parents on using portion guides (Braun and Clarke 2019). See Figure 2 for the analytic process. Themes were developed both deductively, drawing on previous literature and inductively based on patterns observed in the data set (Fereday and Muir‐Cochrane 2006). The analysis was informed by critical constructivist epistemology and contextualist ontology to understand how parents construct their understanding of the portion guide within their individual, experienced contexts (Al‐Ababneh 2020). Interview transcripts were analysed using NVivo 12 (2017).

In line with recommendations for reflexivity, M.M. reflected on her contributions throughout the research process and discussed interpretations with R.C., a female researcher who has children (Braun and Clarke 2023). R.C. double‐coded two transcripts to further consider alternative interpretations. The Consolidated Criteria for Reporting Qualitative Research (COREQ) checklist was used (Tong et al. 2007) to ensure transparency and reporting credibility (see Supporting Information S1: Appendix B).

### 2.4 Ethics Statement

Ethical approval was obtained from the University College London research ethics committee (reference: 21253/003). The participants in this study gave informed consent before completing the study. The privacy rights of human subjects have been observed in the reporting of findings.

## 3 Results

Overall, 137 survey responses were received, of which 33 met inclusion criteria. Of these, 17 responded to emails and were interviewed between June and August 2024. However, two were not included in analysis as new information shared during interviews indicated that they did not meet inclusion criteria. The final sample included 10 mothers and five fathers (Table 1). Participants were aged 27–41 years (*M* = 31.1 years) and represented a range of socioeconomic and ethnic backgrounds. Interviews lasted 27–47 min (*M* = 37 min).

**Table 1:** Participant demographics.

Participant ID	Relationship to child	Age^a^	Ethnicity	Household status	Social grade^b^	Partner's social grade^b^	Gender of child	Age of child in years^c^	No. of children in household	IMD decile^d^
P1	Mother	38	White—non British	Two parent	E	E	Girl	2	2	3
P2	Mother	27	Mixed—White and Black African	Two parent	C1	C2	Boy	1	2	8
P4	Mother	36	Mixed—White and Black African	Two parent	B	C2	Girl	1	1	2
P6	Mother	33	Asian/Asian British—Indian	Two parent	B	B	Girl	4	2	3
P7	Mother	38	Black/African/Caribbean/Black British—Caribbean	Two parent	C1	C1	Girl	3	1	1
P8	Father	37	Black/African/Caribbean/Black British—Any other	Two parent	B	C1	Girl	2	2	7
P9	Mother	33	White—non‐British	Two parent	C1	C2	Boy	2	1	4
P10	Father	41	Asian/Asian British—Indian	Two parent	A	A	Boy	3	2	4
P11	Father	34	Mixed—White and Caribbean	Two parent	B	D	Boy	3	2	1
P12	Father	35	Black/African/Caribbean/Black British—Any other	Two parent	C1	A	Boy	4	2	1
P13	Father	29	Black/African/Caribbean/Black British—Caribbean	Two parent	A	C1	Boy	3	1	3
P14	Mother	36	Asian/Asian British—Indian	Single parent	C1		Girl	3	1	6
P15	Mother	33	Asian/Asian British—Bangladeshi	Single parent	C1		Boy	1	1	e
P16	Mother	31	Asian/Asian British—Indian	Two parent	C1	B	Girl	1	1	6
P17	Mother	31	White—British	Two parent	E	E	Girl	1	2	6

^a^ Mean age of parent = 31.1 years.

^b^ Mean age of children = 2.3 years.

^c^ Social Grade classifications: AB, higher and intermediate managerial, administrative and professional occupations; C1, supervisory, clerical and junior managerial, administrative and professional occupations; mC2, skilled manual occupations; DE, semi‐skilled and unskilled manual occupations; unemployed and lowest grade occupations.

^d^ Index of Multiple Deprivation deciles range from 1 (most deprived areas) to 10 (least deprived areas).

^e^ n/a, participant living in a hotel.

Four themes were developed, focusing on parental portioning strategies, perceptions of the portion guide and the value of portion guidance more widely. See Table 2 for additional quotes from parents.

**Table 2:** Themes and subthemes with additional participant quotes.

Theme 1: Guidance is appreciated but has a limited impact on portions	
1.1. The suggested portions are difficult to use in real life	‘I'm not sure what that means, because in my head it's saying occasionally, then the tip of the thumb, but then on the actual portion size it could be up to four chips or a small piece of cake. […] A small piece of cake wouldn't really fit into a thumb.’ (P4)
	‘With your busy, occupied lives it's very difficult to go back to this chart and take it off whether […] he had five a day or not.’ (P10)
	‘I mean you can have an idea, but really you don't put the food in your hands and, I don't know, I just found a bit difficult.’ (P1)
	‘It's just a bit strange measuring with your hand, I would say.’ (P17)
1.2. Portions do not reflect children's individual differences	‘Most cases they want more than this portion, maybe two times or three times. And here is just telling you to just give your child a little amount. So to me it's not realistic.’ (P7)
	‘So he is a big, tall boy. So he eats a bit more, so I think it should be guided more on child's weight rather than the age because you have different kids, so.’ (P9)
	‘Because obvious and then also people hands are different, right?’ (P1)
1.3. The guide does not show all the foods given to children	‘A vegetarian focused guide as well that would suit more to the families because when I look at this I'll like OK, half of the things are not for me. So probably this is not the right guide for me.’ (P10)
	‘Yeah. So I got the sort of things, other things which I never had before, thinking as my culture is totally different from everything.’ (P15)

Theme 2: Portions are usually determined by other factors	
2.1. Beliefs, knowledge, and experience	‘Use my own, my own personal thinking, OK, this should be enough for for a toddler you know?’ (P13)
	‘My GP actually advised not to give high fat and, you know, a lot of sugar. High sugar actually causes diabetes. […] So I don't. I don't prioritise that.’ (P11)
2.2. The child influences the portions they get	‘Or I use I use his behaviour eating the kind of food I'm cooking if I know he doesn't really like this food and I'm trying […] to give him‐, I kind of reduce the portion I'm I'm giving him.’ (P13)
	‘When she's hungry, like she asks for food, like when she's hungry. She asks.’ (P14)
	‘And I personally feel if child is hungry he comes and tells that he's hungry, he wants to eat something.’ (P10)
2.3. Plates and bowls are useful when deciding	‘I have containers that are different sizes, but then I have a specific one for her.’ (P1)
	‘For those sugary foods. I had a particular plate that I used to measure the amount of sugar food I allowed to take.’ (P2)
	‘So the cues that I've developed now is that I will offer it to her more if if she's completely finished the plate and I'll give her a little bit more.’ (P4)

Theme 3: The guide is still useful, just not as intended	
3.1. Reminder of a balanced diet	‘What I understood from this chart was, I need to have a balanced diet for my child’ (P16)
	‘You try as much as possible to get at least 80% of what is there. But it can be interval not necessarily you have to consume egg and chicken at the same time.’ (P8)
	‘When we make up a plate for her, I just try my best to have a combination of what's on there.’ (P4)
3.2. Moderation of sugar and fat intake	‘It's a reminder to limit the not‐so‐good food for your child. Like if they're eating more than what it says on the guide then obviously you need to limit that.’ (P17)
	‘I don't give too much like you know, even if she cries, it is like half or one piece of cake. That's it. […] It's not the whole thing. Like earlier she used to finish up like 2‐3 and then now it is not like that. Like after looking at this portion size. So it is definitely reduced but not achieve like not achieved as such. Like I'm not following exactly how it's saying.’ (P14)
	‘There have been occasions where he's he's munching cake, you know, more frequently going to more birthday parties. Possibly I'll have a look at this guide and then I'll instruct my wife to not give him a full slice of cake when she's at parties’ (P10)
3.3. Guide inspires mealtimes	‘Yeah, because that helps you so decide my menu what to give her for the week. […] Sometimes our menu is decided by like OK we we can have this for dinner tonight.’ (P16)
	‘The variety is really nice because you can pick different things from each category and put it on their plate and rotate it.’ (P4)

Theme 4: When and how guides are delivered influences acceptability	
4.1. Preferred modality of the guide	‘If it is like a sticker or anything like we can paste it on the wall. So it's easy for us. We'll put it in the kitchen or we'll put it on the fridge. And for us to come and look back.’ (P14)
	‘I guess a pdf would be good. You know something that we could bring up on our phones. And was really busy, really very busy. So anything that's quick like that.’ (P17)
	‘Yes, I think it would be maybe if they would use pictures, probably a plate with the actually food picture.’ (P1)
4.2. There is an appropriate audience and time for portion guides	‘I think it's a perfect one for families that don't have experience of portioning food for the families.’ (P12)
	‘I will say first time parents because for some parent that's the first time experiencing a child's portioning’ (P13)
	‘I think the weaning time when there is time for weaning that time might be the best time to give, because that time the parents will be looking out for more studies and research and all these guides.’ (P14)
4.3. The distributor influences the perception of the guide	‘The medical specialist, it's influenced how the parents are going to use it. It's going to influence their decision of using [the guide].’ (P13)
	‘Your family well‐being centres, where they come for the session, stay and play. Yeah. So it would be a good guide like to give to the parents to follow these.’ (P14)
	‘I think […] libraries are good places because people are quite relaxed in library environment and they have a mindset to gather information.’ (P10)
	‘It's easy to understand. But if somebody you know, tell them that it how it is and what it is, why should you follow it. I think 1/2 an hour session would also help them. You know, it's this more like, you can motivate other people to join, you can motivate people that this will help.’ (P16)
	‘They should try to explain this more to people, especially parents. Not all everybody's educated to understand this.’ (P7)
	‘I think if I actually use her palm and her full hand and a full fist and the fingers to actually measure things out, I might get a realistic idea of what she should be getting. So I definitely want to try and do that in here with her meals.’ (P4)

### 3.1 Theme 1: Guidance Is Appreciated but Has a Limited Impact on Portions

In general, parents were open to receiving portion guidance, many participants indicating that they welcome any feeding or nutrition‐related information. When shown the portion guide during interviews, some parents reported having looked at it occasionally for reassurance that they were generally feeding appropriately. Some parents remarked that they had been surprised by the suggested portion sizes for foods high in fat and sugar, as these were smaller than they would have expected.

Despite initial positive responses regarding the portion guide, when prompted further, parents reported not using the portion guide to determine portion sizes for various reasons, as described below.

#### 3.1.1 The Suggested Portions Are Difficult to Use in Real‐Life

Although parents occasionally looking at the guide for support, they did not routinely use it as feeding situations were often hectic and they lacked the time to consider whether they were serving recommended portion sizes. The guide was seen as an additional burden, especially when the child was requesting a certain amount of food, leading parents to prioritise their child's perceived immediate needs over the guide's suggestions.

They want some food and so at that time it's really difficult to […] measure the portion size and give them the exact portion size mentioned here. […] To make them stop crying we just give whatever they ask for.

(P14)

Additionally, the hand measurements for portions suggested in the guide were perceived as confusing and inconvenient when measuring food portions. Parents also noted discrepancies between the hand‐based portion sizes and the actual food examples provided in the guide.

If somebody says ‘Give a fistful of rice to your child’, I would wonder what does that mean. Do I squeeze my fist or […] ensure that it just lights up?

(P10)

#### 3.1.2 Portions Do Not Reflect Children's Individual Differences

Parents described sometimes using the resource to guide the size of the first portion they served, but then offering more as they felt otherwise their child would be hungry.

What they say it's about a fist of carbohydrates. So that's what we start with. If he feels like eating a bit more then that's fine.

(P9)

However, many parents felt that the guide neglected to consider children as individuals.

This guide is nothing for us, […] because every child is different, his needs are different. Some kids eat much more than my child.

(P10)

Many parents seemed to misunderstand that the ‘How big is a portion guide?’ is intended to be used in relation to the child's hand, so that a bigger child with bigger hands would be given bigger portions. Consequently, they believed the portions suggested in the guide were unsuitable for their child because they were either big or small for their age. This was viewed as a barrier to using the guide, as they felt that a child would end up being either over‐ or underfed if they served the portion that they thought was being suggested.

#### 3.1.3 The Guide Does Not Show All the Foods Given to Children

While most parents felt the food examples represented their child's usual diet, some, for example vegetarians, found the guide more difficult to use because examples did not reflect the foods their family commonly ate.

That is missing a lot because when it comes to protein, it is more of the fish and the meat and chicken, […] which I always have to think […] what to replace it with.

(P16)

I was wondering […] like things like tofu and seitan, […] where would they fall into? Would that be the proteins or…?

(P4)

Some participants of Asian backgrounds mentioned that they would serve foods relevant to their cultures, which were not included in the guide. Parents had to then think about what foods in the guide they would compare those cultural foods to, making it challenging to estimate appropriate portions.

Instead of the beans and all, we can have dahl and for the sugary kind we'll be adding instead of cake, sweets.

(P6)

### 3.2 Theme 2: Portions Are Usually Determined by Other Factors

While participants did not generally use the portion guide to determine portion sizes, they reported using a variety of strategies to guide their decisions.

#### 3.2.1 Beliefs, Knowledge and Experience

Many parents relied on their own feeding experiences and explained that they knew their children well and were aware of how much food they usually ate.

It's just for you to use your own experience and the knowledge you've acquired from experience. Experience is the best teacher, I would say.

(P12)

Some parents reported using their portion size as a guide when thinking about an appropriate portion for their child.

I would give him about one‐fourth of what I'm eating. And trying to get more protein than the starchy food […]. But about one‐fourth of what I was eating.

(P9)

The perceived healthiness of the food also influenced portion sizes. Parents did not generally feel the need to restrict portions of vegetables and fruits, whereas foods high in fat and sugar were perceived as unhealthy and therefore something they should try to serve in smaller portions.

In addition to their personal beliefs, participants acknowledged that advice from taking part in the HFRFTS programme or other parenting courses and advice from healthcare professionals contributed to their understanding of appropriate portion sizes. Even though participants were not following the specific guidance on portion sizes, they reported having gained important knowledge from these sources.

I was not sure because I felt it's very less quantity, how will it fill her stomach? But then when we went to the HENRY programme, I got to know that their size of stomach is only this much.

(P16)

#### 3.2.2 The Child Influences the Portions They Get

Parents reported closely monitoring their children and observing their responses to foods and portions. Foods preferred by the child were served in larger quantities, especially when the child had a small appetite.

For the milk and dairy, I give him more because that is what he loves to take. […] So I give him more […] than what was listed above in the guide.

(P13)

Some parents mentioned satiety cues and discussed not wanting to force their child to eat or leave them hungry, but trying to respond to the child's behaviour.

If she's not satisfied, she cries. […]. She starts knocking on the plate […] and you know that she wants more. And if she's OK, you see how she just drops the plates on the floor.

(P7)

#### 3.2.3 Plates and Bowls Are Useful When Deciding

Several parents indicated that child‐specific tableware, such as child‐sized bowls and plates, served as practical tools for portioning, particularly for lunch and dinner.

I've got her small cups […] that she should finish that much, and I'll be like now she's done.

(P16)

### 3.3 Theme 3: The Guide Is Still Useful, Just Not As Intended

Despite the portion guide rarely being used to guide portion sizes served, parents still perceived it as a helpful resource.

#### 3.3.1 Reminder of a Balanced Diet

Many parents felt that the guide served as a helpful reminder of the importance of having a diverse diet. They elaborated that if they had served one food group during an earlier meal, the guide reminded them to offer food from the other food groups at subsequent meals. Parents felt that if not reminded of the importance of a diverse diet, they might over‐serve some food groups.

Because of this guide we at least follow, OK potato is not in the vegetable, we need to give some vegetables. We need to give different food groups […] so it's really helpful in that way.

(P14)

#### 3.3.2 Moderation of Sugar and Fat Intake

Parents also used the guide to limit their child's sugar and fat intake. They felt that the red background for high‐fat and high‐sugar foods reminded them to avoid serving them in excessive amounts. However, some felt that including less healthy foods in the guide could be counterproductive, as it could be interpreted as indicating they are a necessary part of children's diets.

It's in a red colour, so it's good to have because red colour we understand [that] red means something dangerous. They can have, but it's something dangerous.

(P15)

#### 3.3.3 The Guide Inspires Mealtimes

Another way parents were using the guide was to get examples of foods they could serve their children. Participants explained that looking at the foods listed in the guide, could help them decide which foods to include in meals. Therefore, they felt it was important that example foods were healthy foods.

Sometimes I'm thinking what am I going to cook for dinner today, and maybe we'll even get ideas what to cook [from the portion guide].

(P1)

Some felt the guide could be more helpful and inspirational. They appreciated visual aspects, such as different colours and pictures, but, some felt that seeing more pictures or photographs of foods would aid their understanding.

It's nice and colourful. I'm just wondering maybe if like realistic photos of the food would be helpful for some people […] if there's a language barrier.

(P4)

### 3.4 Theme 4: When and How Guides Are Delivered Influences Acceptability

Parents suggested how the guide should be delivered to make it as useful as possible.

#### 3.4.1 Preferred Modality of the Guide

Parents emphasised the need for portion guidance to be accessible. For some, this meant providing the guide as a leaflet to stick on the fridge, others favoured online resources accessible by phone. Some wanted the guide as a PDF in their emails, so that they could look at it when needed and/or print it out.

#### 3.4.2 There Is an Appropriate Audience and Time for Portion Guides

Although participants felt that all parents would benefit from receiving the guide, many felt that first‐time parents would be particularly receptive. Participants reflected that when they had their first child, they lacked the knowledge and confidence to make portion decisions, making them more open to advice and more likely to use the portion guide.

I think first time parents will really benefit from this because they are exploring. […] everything is new to them. They don't know what is the right size to feed their babies.

(P10)

Parents believed it was important to receive age‐appropriate portion information before their child started solids or during the early stages of weaning when they were establishing regular eating patterns. Additionally, participants mentioned that with older children feeding patterns and practices have already been established, meaning that a guide might have less of an impact.

#### 3.4.3 The Distributor Influences the Perception of the Guide

Several parents mentioned trusting information provided by their General Practitioner (GP) and health visitor and that they would be appropriate professionals to provide portion guidance. They believed information from healthcare professionals was perceived as important for health and they paid more attention to trying to put it into practice. Others felt places like children's centres, health centres and libraries were ideally placed to provide a portion guide as parents regularly attended these with their children.

Definitely by the health visitor, because we can rely to the health visitor more than the other people. […] I tried to follow everything my health visitor tells to me.

(P15)

#### 3.4.4 Simply Handing out a Leaflet Is Not Enough

Although participants welcomed nutrition guidance, they said they might discard or forget a leaflet that was just handed out. It was generally felt that parents would be more likely to use a portion guide if someone provided an explanation and advice about using it, as happens with HENRY.

But by giving to the parents […] a quick explanation, parents will be interested and maybe they'll take it home and read it and go through it.

(P1)

It was felt that a verbal explanation strengthening parents’ motivation to use the guide, which was demonstrated during the interviews themselves. Several parents reported not having used the guide since receiving it but at the end of the interview, they all felt they had gained a better understanding of the guide and intended to use it moving forward.

## 4 Discussion

This study was the first to explore parents' experiences and beliefs on using portion guidance after receiving an age‐appropriate portion guide for young children. While parents generally appreciated receiving the guide, they found it impractical to apply in real‐life situations. Instead, they relied on a range of parent‐ and child‐led strategies to decide portions. Participants felt the guide prompted them to serve a more balanced diet and provided ideas about suitable foods. They believed first‐time parents in particular would benefit from accessible portion guidance from reliable sources.

The gap between the intended purpose of the guide and its practical application is consistent with a recent study by Quirke‐McFarlane et al. (2024), in which a small proportion of parents reported being vaguely aware of portion guides but felt they would be impractical to use. Similarly, parents of older children (8–11 years) have indicated that they would not welcome portion guides as they felt it was impractical to measure the amount of food they served children (Croker et al. 2009). This suggests that although parents of young children have expressed interest in receiving a portion guide (Martin‐Biggers et al. 2015; Reale et al. 2019; Philippe et al. 2021), when receiving one it might guide their portioning practices less than anticipated due to the realities of child feeding.

Previous research indicated parents often use their child's hand size as a practical guide for estimating age‐appropriate portion sizes (Blake et al. 2015; Reale et al. 2019), as recommended by the Early Years Foundation Stage nutrition guidance (Department of Education 2025). However, parents in the current study felt that the hand measurements suggested in the HENRY guide were impractical. Perhaps hand measurements are intuitive for certain foods, but formalising the process and applying it to all foods is inappropriate. One issue reported in the current study was that the hand measurements presented did not correspond to the example food portions. An alternative way of presenting portion information might be in the form of a plate model similar to the Eatwell Guide (NHS 2022) or with pictures of food items served on a plate, as is done in some existing guides (First Steps Nutrition Trust 2016; British Nutrition Foundation 2019).

The types of foods shown in ‘How Big is a Portion?’ appeared to limit its utility for some parents, especially those followed a vegetarian or culturally specific diet, which aligns with previous research with minority ethnic groups in high‐income countries (Ojo et al. 2023). Evidence suggests some ethnic minorities may also be reluctant to follow feeding recommendations, partly due to inconsistencies between the information provided by healthcare workers and family members (Manikam et al. 2023). Culturally tailored support for using the portion guide, including alternative food examples, is given to parents attending the 8‐week HFRFTS programme. However, course attendance was not measured in the current study, and it is unclear whether parents attended or could recall this guidance. Findings reinforce recommendations from NICE to provide feeding support tailored to individual circumstances (National Institute for Health and Care Excellence [NICE] 2025). Future research could explore the utility of providing portion guidance in an app or employing Artificial Intelligence (AI) technologies to facilitate individual tailoring.

Parents described employing similar child‐ and parent‐driven strategies for deciding portions as parents who have not received a portion guide (Porter et al. 2023; Quirke‐McFarlane et al. 2024). As reported in previous studies, child‐size tableware was considered helpful (Porter et al. 2023; Reale et al. 2019), and there was a reliance on personal experience and knowledge (Johnson et al. 2015). Similarly, participants felt it was important to recognise that every child is different (Porter et al. 2023). They reported offering children more food if requested, in line with previous research (Vidal et al. 2024) and the principles of responsive feeding. Responsive feeding is an integral part of the HFRFTS programme, which supports responding to hunger and satiety cues rather than encouraging children to eat predetermined amounts of food (DiSantis et al. 2011; Daniels et al. 2012). Parents of children with avid appetites sometimes feel unsure about how to respond to frequent requests for food and additional portions, and may find a portion guide helpful for balancing their child's diet and encouraging them to consume fruit and vegetables rather than energy‐dense snacks (Edwards et al. 2024).

Several participants mentioned that the way the portion guide was presented helped remind them to serve a more balanced and diverse diet, rather than providing specific portion sizes, a finding consistent with previous literature (Croker et al. 2009; Goldthorpe et al. 2018). They consequently used the portion guide to remind themselves of food groups and to inspire food choices. However, parents following a vegetarian or vegan diet found the example foods limited, especially in the ‘protein’ category. This highlights the importance of presenting healthy and sustainable options in portion guides and raises concerns about other portion guides which have been developed with industry partners and show images of highly processed foods, including energy‐dense snacks and confectionary (More and Emmett 2015; Infant and Toddler Forum 2019). Additionally, this reiterates the need for healthy examples to be culturally representative if they are going to inform feeding decisions.

Parents described being receptive to learning about children's nutrition, but emphasised the importance of information being clear, practical and actionable. They felt that examples, such as one tablespoon of ice cream, helped them limit their child's sugar and fat intake. This aligns with previous research suggesting parents are willing to limit portion sizes of foods seen as unhealthy (Martin‐Biggers et al. 2015; Sherry et al. 2004). As consuming foods high in fat and sugar is associated with an increase in childhood BMI (Millar et al. 2014), providing portion guidance for these foods is particularly relevant. Some participants questioned whether including high fat or sugar foods in the guide might be interpreted as indicating they are a necessary part of a child's diet. However, in line with previous research (Porter et al. 2023), many parents felt it was helpful to include high fat and sugar foods while highlighting that intake should be limited. Presenting high fat and sugar foods in a separate section was considered a helpful reminder that they should not be considered in the same way as more nutrient‐dense foods.

Although SACN recommend developing age‐appropriate portion guidance, findings suggest portion guides may not be used as intended (Scientific Advisory Committee on Nutrition [SACN] 2023). Future research could investigate alternative strategies parents might find more practical to implement in daily life, such as visual aids, meal‐planning tools, and providing clearer labelling on snacks. As parents found the portion guide helpful for balancing their child's diet, future research could explore developing more holistic tools to support feeding rather than focusing on portion sizes in isolation. Future studies should also aim to understand the information needs of parents from subgroups to develop more inclusive and culturally sensitive resources.

Participants' trust in feeding guidance provided by health professionals aligns with previous research (Spyreli et al. 2021). Parents from lower socioeconomic backgrounds have been found to trust dietary advice from doctors (Slusser et al. 2011), which highlights the importance of improving nutrition education in medical schools, as it is often extremely limited (Jones et al. 2023). Findings from our study and others, including Porter et al. (2023), point to the benefits of providing feeding support for first‐time parents in particular, as a part of routine appointments or at sessions in children's centres. National Institute for Health and Care Excellence [NICE] (2025) recommend considering individual circumstances when providing support for families and our findings highlight the importance of tailoring advice to individual dietary and cultural circumstances, as well as children's food preferences and appetitive traits. Individualised support can help facilitate healthier dietary intake from early childhood and improve parents' confidence and feeding behaviours (Willis et al. 2016).

A limitation of the current study is the relatively small sample size, and it is acknowledged that people who volunteer might be especially receptive to feeding information. Additionally, as the study focused on a single portion guide, it was not possible to explore the features of other guides that caregivers may or may not find helpful. The current study overcame limitations related to lack of diversity reported by previous researchers (Kairey et al. 2018; Porter et al. 2023) by including both female and male participants from multiple ethnic backgrounds. The authors acknowledge that their professional and personal experiences may have influenced the analysis, as both M.M. and R.C. have prior knowledge of feeding recommendations, and R.C. has personal experience in feeding children. However, both authors contributed to the research design and data analysis, ensuring that differing opinions were considered and R.L. was not involved in the analysis.

## 5 Conclusion

This is the first study to examine parents' experiences of using a portion guide when feeding their children. Parents provided with the HENRY portion guide did not tend to use it when deciding portion sizes to serve but they welcomed it in terms of adding diversity to children's diet, limiting unhealthy foods and providing ideas for mealtimes. Several aspects of the portion guide were considered confusing, and it was perceived as difficult to use when faced with the realities of child feeding. When assessing the value of making age‐appropriate portion guides more widely available, it was discovered that participants were open to receiving information on children's nutrition from sources they deemed reliable. Findings highlight the importance of considering the realities of parenting when developing and distributing resources, as well as providing parents with opportunities like HFRFTS to access support for feeding their children.

## Author Contributions

**Mira Malmberg:** writing – original draft, methodology, investigation, formal analysis, data curation. **Rebecca Lang:** writing – review and editing. **Rana Conway:** writing – review and editing, methodology, supervision, conceptualisation.

## Conflicts of Interest

The authors declare no conflicts of interest.

## Supporting information


**Appendix A:** Interview schedule for the semi‐structured qualitative interviews. **Appendix B:** The COREQ checklist (Tong et al. 2007) used to maintain transparency and to ensure the credibility of reporting throughout this manuscript.

## Data Availability

The data that support the findings of this study are available from the corresponding author upon reasonable request.
