# Supplementary material for: Understanding Parents' Experiences of Using a Portion Guide for Young Children: A Qualitative Study
Source: Matern Child Nutr. 2025 Dec 13;22(1):e70151. doi: 10.1111/mcn.70151 (PMC12701707; doi:10.1111/mcn.70151)
Supplement: Supplementary file 1 — Appendix A: Interview schedule for the semi‐structured qualitative interviews. Appendix B: The COREQ checklist (Tong et al. 2007) used to maintain transparency and to ensure the credibility of reporting throughout this manuscript. [file MCN-22-e70151-s001.docx]

**Supporting Information for: Understanding parents’ experiences of using a portion guide for young children: A qualitative study**

**Appendix A**

*Interview schedule for the semi-structured qualitative interviews.*

[General introduction of the interviewer and aims of the research. At this point the participant is given the opportunity to ask any questions before the interview and recording of the interview begins]

Part A. General decision making around feeding children

1. First of all, could you tell me a bit about your baby’s/child’s appetite and how you found weaning? (fussy eater, big/small appetite, favourite foods)
2. Could you tell me more about the kind of foods your son/daughter eats now? (pouches, family meals) and what meal times look like? (who else is eating, where sitting)
3. When you first started introducing solid foods to your child, how did you decide how much food to give your baby? (any foods easier/less easy to decide about)
4. How about now - has the way you decide about how much food to give your child changed as they have got older?

Are there any foods that are easier/harder to decide about now?

Part B. Explore perceptions of the HENRY guide

The HENRY portion guide will be shown by sharing a screen with the participant and the following questions will be asked about the guide.

1. I’m interested in what parents think about this portion guide (show ‘How big is a portion’). Could you tell me if you remember being given it?

How were you introduced to it? (by who, baby’s age, what were you told about it, what did you do with it, did anyone else see it/use it e.g. dad, granny)

1. Have you used the guide when deciding how much food to give your child? (did usage change over time)

If so, could you tell me more about how you have used it?

If not, what are some of the reasons you have not used it?

1. How useful do you find the guide when making decisions about your child’s diet and portion sizes?

Why do you think this is?

1. Let’s look at the columns titled “1 and 2-year-olds” and “3 and 4-year-olds” more closely. How realistic do you feel like the portion sizes highlighted in those columns are when you consider what your child eats now?
2. Focusing on those columns still, how do you feel about the examples shown?

Are there any foods you feel like are missing and should be included?

Are any of the examples irrelevant in real-life?

How important are the examples for

1. The foods at the bottom are foods that young children don’t need and most children eat too much of these. How clear do you think that is the way it’s presented at the moment?

Can you think of any way of making it clearer? (might it be better not to include these foods, since they’re not recommended?)

1. If you were telling another parent about how to use the HENRY guide, what would you say?
2. What do you like about the guide?
3. If you were asked to make it more useful for parents, what changes would you make? (images/photos, age groups shown,)

Why would you like this to be changed?

Part C. Explore the preferred modality of the HENRY guide

1. Do you think being given the portion guide in paper format is useful for parents?

Why/why not? (where did they put it, was it suggested that you put it on the fridge or somewhere else, do you think it would be helpful to suggest parents put it on the fridge/somewhere else, does it matter if it’s paper/card/laminated)

1. Is there a form of guide that you feel could be more useful? (App, Website, why)
2. Could you tell me your thoughts about where it would be the most useful for parents to receive the guide from? (doctors, public sources etc.)

Do you think the place that offers the portion guide would influence parents’ views on it? (e.g. a company or doctor)

1. Who might find the guide most useful? (first time mums, dads, grandparents, nursery)

Part D. Any other thoughts about the HENRY portion guide

1. (If the participants said they have thrown the guide away and not really looked at it) Having now taken part in this interview and looked at the guide in more detail, has your view about using it to guide feeding your child changed?

(How/why?)

1. Is there anything else you would like to say about feeding your child or the HENRY portion guide that I have not asked in this interview?

**Appendix B**

*The COREQ checklist (Tong et al., 2007) used to maintain transparency and to ensure the credibility of reporting throughout this manuscript.*

*
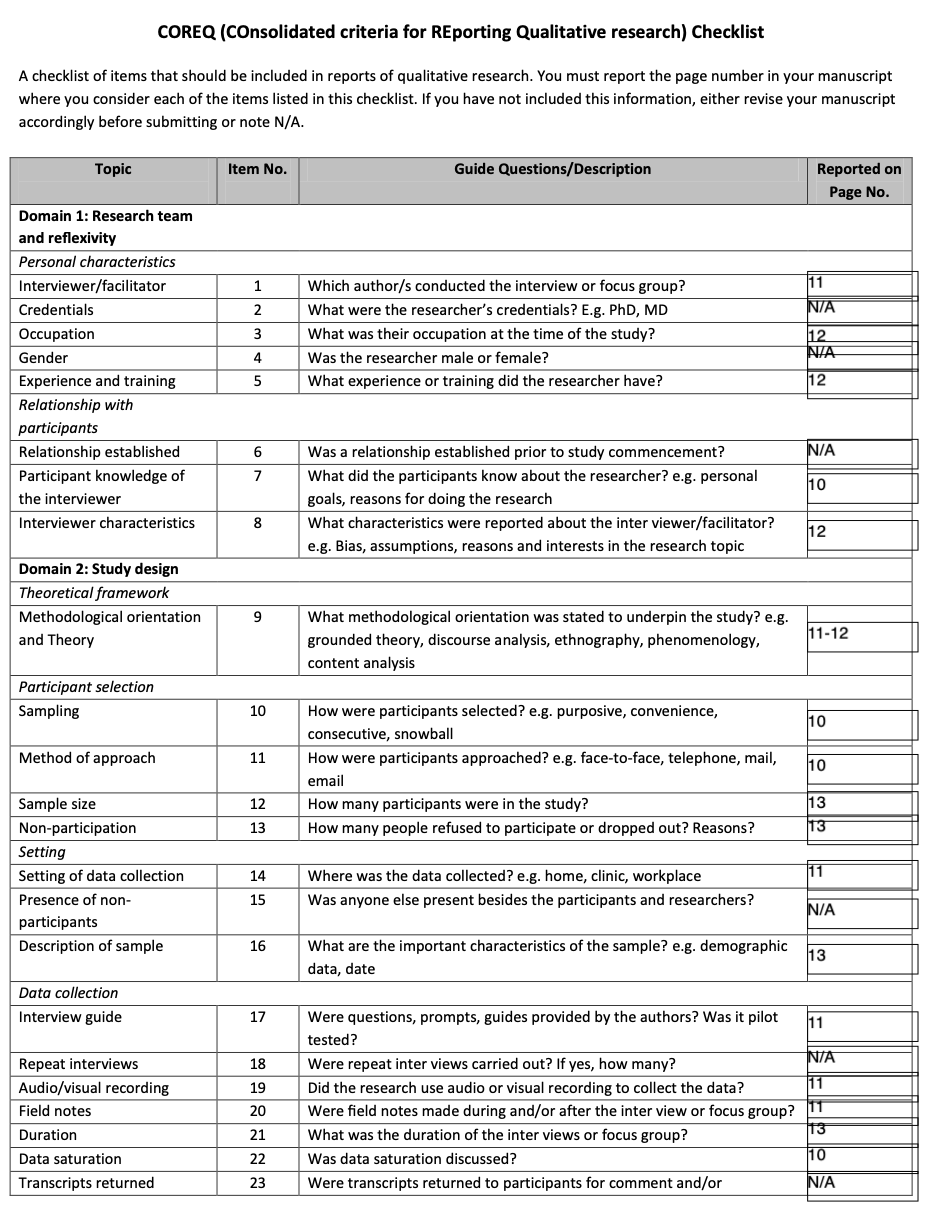
*

*
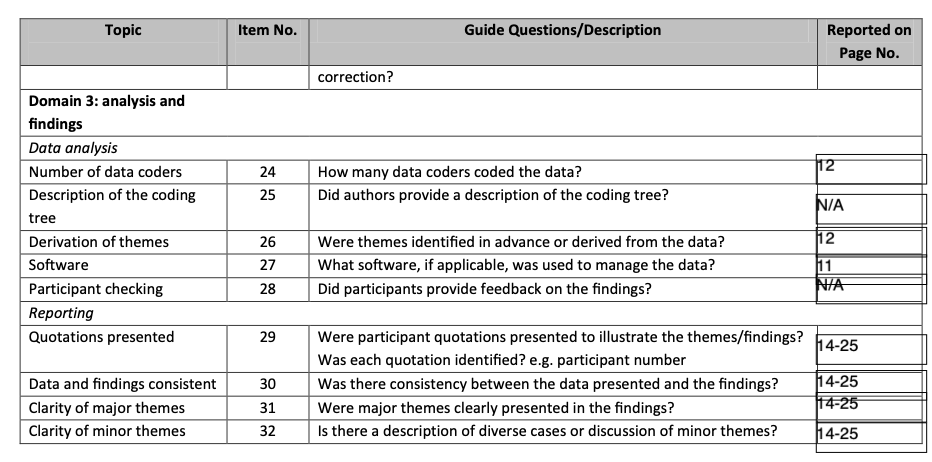
*
